# Traditional Neuropsychological Testing Does Not Predict Motor-Cognitive Test Performance

**DOI:** 10.3390/ijerph17207393

**Published:** 2020-10-11

**Authors:** Jan Wilke, Oliver Vogel, Sandra Ungricht

**Affiliations:** Department of Sports Medicine & Exercise Physiology, Goethe University Frankfurt, 60488 Frankfurt am Main, Germany; vogel@sport.uni-frankfurt.de (O.V.); s.ungricht97@googlemail.com (S.U.)

**Keywords:** neurocognition, reaction, vision, sports performance

## Abstract

The ecological validity of neuropsychological testing (NT) has been questioned in the sports environment. A frequent criticism is that NT, mostly consisting of pen and paper or digital assessments, lacks relevant bodily movement. This study aimed to identify the determinants of a newly developed testing battery integrating both cognitive and motor demands. Twenty active individuals (25 ± 3 years, 11 males) completed the new motor-cognitive testing battery (MC), traditional NT (Stroop test, Trail Making test, Digit Span test) and isolated assessments of motor function (MF; Y-balance test, 20m-sprint, counter-movement jump). Kendal’s tau and partial Spearman correlations were used to detect associations between MC and NT/MF. Except for two items (Reactive Agility A and counter-movement jump; Run-Decide and sprint time; r = 0.37, *p* < 0.05), MC was not related to MF. Similarly, MC and NT were mostly unrelated, even when controlling for the two significant motor covariates (*p* > 0.05). The only MC item with (weak to moderate) associations to NT was the Memory Span test (Digit Span backwards and composite; r = 0.43–0.54, *p* < 0.05). In sum, motor-cognitive function appears to be largely independent from its two assumed components NT and MF and may represent a new parameter in performance diagnostics.

## 1. Introduction

Fitness testing represents an essential component of conditioning in sports [1,2,3,4]. Particularly, markers of motor function (e.g., upper-body strength, sprint speed, jump height), represent popular outcomes frequently used to gauge the success of athletes and teams [2]. However, recent investigations have questioned the ecological validity of related assessments in game sports. Nightingale et al. [3] reviewed the value of classical off-ice motor testing in ice hockey. They concluded that the collected data, in most cases, were not or only weakly correlated with sports performance. Teramoto et al. [4] made similar observations examining motor assessments in elite basketball athletes. Contrary to anthropometric factors such as height or wingspan, which weakly predicted sports-related success, only small or no associations at all were found for markers of motor function [4]. Both examples demonstrate that movement proficiency, captured in isolation from game sport demands, represents a facet, but by far not the only determinant of performance in many interactive sports.

Taking into account the limitations of conventional performance tests, recent research has sought to identify alternatives supplementing the current diagnostic spectrum. A plethora of studies focused on the potential role of perceptual-cognitive function [5,6,7,8,9,10]. Briefly, it has been suggested that superior levels of related skills (e.g., visual scanning, attention, working memory, inhibitory control) could help to increase sports performance and prevent injury [6]. The rationale behind these claims is that engaging in game sports mostly requires open vs. closed skill performance, not allowing an exclusive reliance on pre-planned motor actions, such as deciding to run, jump or cut into one direction without changing the original plan. In contrast, athletes interact in a highly unpredictable and time-constrained environment, reacting to external stimuli such as the opponent(s)’ and/or team mate(s)’ positions or the movement of the ball. The ability to quickly process the multifaceted afferent input in the brain could thus (a) represent a decisive criterion from a success- and health-oriented perspective and (b) explain the weak correlations between sports performance and isolated assessments of motor function [11].

Due to the arguably high relevance of perceptual-cognitive function in game sports, several researchers have included neuropsychological testing when designing studies in sport-and movement-related settings [10]. Notwithstanding, the question of ecological validity does also apply here because the chosen assessments in most cases were pen and paper or computer-based, thus lacking major bodily movement. For instance, the Stroop test, a measure of attention, processing speed and inhibitory control, requires the participants to read congruently and incongruently written names of colors (e.g., red written in red as opposed to red written in blue, [12]). The Trail Making Test targets visual scanning, working memory cognitive flexibility by means of connecting randomly arranged numbers and/or letters in a specific order [13]. Interestingly, several studies, particularly in football, show that associations exist between sports performance and specific domains of cognitive function [9,10]. However, these data do not allow assumptions on causality and may describe a translucent correlation: The inference that athletes act more efficiently on the field because of performing well in such neuropsychological tests may hence be as incorrect as the assumption that sprint speed measured without contextual factors (e.g., reacting to opponents, team mates or ball movement) will directly transfer to in-game performance. 

In a previous trial, we have presented a motor-cognitive testing battery, which aims to concurrently assess cognitively challenging tasks and functional upper- and lower-limb movement [11]. While high reliability was demonstrated, it is unclear which determinants predict test performance. Specifically, it is unknown whether the contributions of traditional neuropsychological testing and motor function testing (e.g., explosive strength) are balanced or if motor-cognitive action outcomes are dominated by one of both. The present study addressed these questions revealing the correlation between neuropsychological testing and motor-cognitive testing after controlling for relevant markers of motor performance. 

## 2. Materials and Methods

### 2.1. Ethical Standard

A cross-sectional study, approved by the local ethics committee, was conducted according to the Declaration of Helsinki as well as the guidelines of Good Clinical Practice. All enrolled participants provided written informed consent. 

### 2.2. Sample

Twenty healthy sports students (24.8 ± 2.5 years, 11 males) volunteered to participate. Exclusion criteria comprised deficiencies in color vision, severe orthopedic, cardiovascular, neurological, endocrine diseases, psychiatric disorders, acute inflammation or history of surgery in the lower limb, intake of drugs that modify pain perception and proprioception, muscle soreness and pregnancy or nursing period. Recruitment was performed by word of mouth. 

### 2.3. Measurements

All participants were tested for three groups of outcomes: motor-cognitive function (MC), neuropsychological testing (NT) and motor function (MF). All participants visited the laboratory twice with a five-to-seven-day interval between. In the first session, we examined markers of MF. Additionally, a familiarization with the disposed NT assessments was performed to prevent practice effects [14]. The second session served for the actual NT assessments and, after a 1-h break, MC testing.

### 2.4. Motor Function

Assessments of MF comprised markers of dynamic balance, strength and explosive force capacities. For *dynamic postural control*, the Y balance test was performed [15]. Standing on one leg, the participants were required to push a slider on the ground as far as possible into the anterior, posteromedial and posterolateral direction with the foot of the free leg. Three repetitions were performed for both sides and each direction, respectively. They were classified as correct if the standing leg did not move, the hands were maintained at the pelvis and the free leg was moved at a constant speed without loss of contact to the slider. From all six movements, the composite score, which has been shown to be highly reliable (ICC: 0.91 to 0.99, [15]), was calculated as (anterior + posteromedial + posterolateral distance) × 100/3 × leg length.

Two methods were used to assess *strength* and *explosive force* capacities. Firstly, 20 m sprints (3 repetitions with 1-min rest intervals, best trial used for analysis) were performed. The time needed is strongly associated to rate of force development and change of direction speed [16,17]. It was measured by means of a light-based sensor system (FitLight, Fitlight Corp., Aurora, ON, Canada). Sprint assessments are highly reliable (ICC: 0.88 to 0.98, [18]). Secondly, counter-movement jump (CMJ) height was measured using a contact mat (Refitronic, Schmitten, Germany). The participants were instructed to perform three repetitions (best trials used for analysis), keeping their hands at the pelvis. The CMJ has been demonstrated be a strong predictor of explosive power and exhibits excellent reliability (ICC: 0.98, [19]). While sprint performance correlates strongly with the initial phase explosive force capacities, CMJ height is the strongest predictor of force production in the late phase [20]. Both methods hence complement each other as markers of explosive strength related to game sport performance.

### 2.5. Neuropsychological Testing

Three assessments were performed to estimate general cognitive function at rest. The *Stroop test* has three parts. In the first and second, capturing attention, the participants had to name the words written or colors shown on a sheet as quickly as possible. The third part of the Stroop test represents a measure of inhibition control. Words of colors were listed incongruently (e.g., “green” written in red or “blue” written in yellow). The participants had to state the color of the word while ignoring its semantic meaning. In all tests, time [s] needed to complete the task was recorded. The Stroop test has been demonstrated to display high reliability (ICC: 0.80 to 0.97, [21,22] and internal consistency (Cronbach’s alpha: 0.93 to 0.97, [22]). 

The *Trail Making test* (TMT) consists of two parts. In part A, the participants were required to connect linearly increasing numbers at maximal possible speed using pen and paper. In part B, successive numbers and letters (e.g., from 1 to A to 2 to B) were to be linked alternatingly. Similar to the Stroop test, time needed for completion was recorded. The results of the test provide a measure of visual screening/attention (TMT-A) and cognitive flexibility/working memory (TMT-B). High reliability and construct validity of the TMT have been shown [13]. 

In the *Digit Span* test, two conditions were also performed. In the first, the participants had to memorize and repeat increasing amounts of numbers read out to them. At the beginning, four numbers were to be recalled. In case of successful memorization, five numbers were named. For each step, two repetitions were performed and one or zero points were awarded depending on recall success. The test ended if both trials failed. The second condition of the Digit Span test was identical to the first but the numbers had to be repeated in reversed order (e.g., 2,4,7,9 becomes 9,7,4,2). While repeating forwards (part 1) captures normal memory, repeating backwards (part 2) is a surrogate of working memory. In addition to the two sub-scores, a composite score, summing both of these, was computed as an overall measure of memory capacities. The reliability of the Digit Span has been demonstrated (ICC: 0.80, [23]). 

### 2.6. Motor-Cognitive Function

Motor-cognitive testing, characterized by cognitive engagement during motor activity, was performed using the FitLight Trainer system (Fitlight Corp., Aurora, ON, Canada). It consists of a control tablet and eight circular wireless sensors equipped with LED lights. At a maximum distance of 50 m to the tablet, the sensors (diameter: 10 cm) can be positioned freely on the ground or attached to objects such as cones or walls according to the design of the planned intervention or test. The use of the system is based on the deactivation of the lights (yellow, green, red, dark blue, light blue, violet) shown by the sensors. Deactivation of lights is achieved by proximity (swiping above the sensor). 

A full-detail description of the testing battery can be obtained in a previous paper [11]. Briefly, the instrument includes six parts (Table 1), three of them for both the upper and lower extremity. Together, the battery items aim to capture typical cognitive and motor demands of game/interactive sports. The first four tests (Reaction, Choice-Reaction, Stop-Signal, Memory Span) include basic motor actions in rather controlled positions. The other two (Reactive Agility, Run-Decide) combine more complex cognitive skills and athletic movement patterns. For instance, in the Reactive Agility test, the participants, inter alia, require explosive acceleration, fast lateral movement and change of direction skills to navigate between the cones as well as effective visual search, working memory and cognitive flexibility to identify the next target to approach. In the Run-Decide test, cutting maneuvers need to be performed during running and their success is dependent on attention and inhibitory control. Together, the mentioned cognitive and motor skills, arguably being central to time-constrained movement on the field, have been suggested to play an important role in sports performance and injury prevention [3,4,9,24].

### 2.7. Data Processing and Statistics

Due to violations of the normality assumption, which was examined by means of the Shapiro–Wilk test, non-parametric methods were used for data analysis. Initially, potential MF confounders of the association between MC and NT were identified. This was done by calculating Kendall’s tau correlations between the individual MC tests and those MF parameters that may plausibly influence them (Table 2). If (a) no significant effects were detected, correlations were then computed for MC and NT wherever a relation between both was plausible (Table 2). If (b) a significant association between MC and MF was detected (potential confounding), partial Spearman rank correlations were performed revealing the relation between MC and NT while controlling for the significant MF factor. The significance level was set to α = 0.05 for all analyses. According to Evans [25], correlation coefficients were graded as poor (<0.2), weak (0.2 to 0.4), moderate (0.4 to 0.6), strong (0.6 to 0.8) or optimal (>0.8). All calculations were made with BiAS for Windows 11.2 (Goethe-University, Frankfurt, Germany).

## 3. Results

All participants completed the experiment and no injuries or other adverse events occurred. 

### 3.1. Relation of Motor-Cognitive Function and Motor Function

MC was not associated with MF in the majority of the tests (*p* > 0.05). However, for the items Reactive Agility A and Run-Decide, weak correlations were found with CMJ and sprint time, respectively (both r = 0.37, *p* < 0.05). 

### 3.2. Relation between Motor-Cognitive Function and NT

Similar to the relation with MF, MC was unrelated to NT in most cases (*p* > 0.05). Correlations were only found for the Memory Span test (DS backwards: r = 0.54, *p* = 0.02, DS composite: r = 0.43, *p* = 0.01, trend for TMT-B: r = 0.31, *p* = 0.06) and the revised order condition of the Run-Decide test (r = −37, *p* = 0.03). The latter association, however, vanished after controlling for the significant motor covariate (sprint time, *p* = 0.08).

## 4. Discussion

The present study provides initial data suggesting an independency of motor-cognitive testing from isolated motor/cognitive assessments. This means that the ability of athletes to effectively perform fast motor actions under time constraints may not be efficiently predicted with usual testing paradigms. The lack of an association between motor-cognitive and purely motor or cognitive skills could be due to the motor-cognitive interference phenomenon [26]: If complex movement is combined with the simultaneous engagement in tasks requiring mental resources, performance decreases in one or both can be observed, depending on difficulty and degree of task automatization. For instance, Fait et al. [27] examined the mutual impact of concurrent cognitive and motor activities in elite ice hockey players. Increasing sport-specific motor task complexity induced gradual losses in cognitive performance while imposing higher cognitive loads reduced sport-specific motor performance. Similar findings were made by Talarico et al. [28]. The authors observed lower squat depths and speeds with simultaneous application of the Stroop or Brooks paradigm when compared to the motor-task only. In the same way, cognitive reaction performance decreased with concurrent motor activity in relation to a singular task without bodily movement. Contrary to the examples provided above, no cognitive-motor interference seems to occur in less multidimensional sports. Having to perform a Stroop task has been shown not to affect time, pacing or cadence in a rowing time trial [29]. Considering that rowing represents a highly automated movement pattern with limited environmental demands and arguably does not require significant cognitive control when compared to team sports, this result seems plausible. 

As many athletes in game sports face complex integrated cognitive-motor demands, identifying the magnitude of dual-task-related performance decrements could substantially amend the current testing canon which largely focusses on isolated/single-task assessments [3]. We hypothesize that athletes, while being on a similar level in isolated tests of motor or cognitive function, may exhibit substantially different magnitudes of cognitive-motor interference. Initial experimental data support this assumption. Gabbett, Wake and Abernethy [30] examined the ability of rugby players to perform a typical motor drill under single-task (drill only) and dual-task (drill and verbal tone recognition) conditions. While no differences were found between both groups regarding the single-task conditions, high-skilled athletes were able to reduce the performance decrease in the dual-task condition when compared to less-skilled athletes. With a similar design, Schaefer and Scornaienchi [31] tested amateur and elite table tennis players by means of an isolated working memory task as well as by means of a dual-task combining the cognitive test with a sport-specific motor component. While the dual-task caused working memory performance deficits of up to 50% in the amateur athletes, the experts experienced a 10% drop only. 

Some limitations need to be discussed. Although our study had sufficient power to detect weak to moderate associations, a larger sample size may have been warranted to detect correlation of smaller magnitude. Additionally, due to the explorative character of our investigation, we did not perform an alpha error adjustment. Possibly, controlling for test inflation would have further reduced the number of significant findings. When assessing isolated motor function, we decided for sprint time, the counter-movement jump and the Y-balance test. These variables were chosen because the underlying constructs, particularly explosive strength, force production and dynamic postural control are suggested to play a major role in most team game sports [2]. We acknowledge that far more tests of motor function (e.g., strength testing with dynamometers, flexibility assessments) are available and applied in practice [1,3,4]. However, firstly, it would have been impossible to implement all these in the present study, and secondly, they almost all share the characteristic of not including significant cognitive affordances. The same applied to neuropsychological testing. Again, with the Stroop, Trail Making and Digit Span test, we chose highly used methods, but it may be possible that other neuropsychological tests would have correlated with cognitive-motor performance. While we doubt this for similar reasons as outlined above (lack of athletic motor components), it should be noted that future studies with other general cognitive or motor tests may still be worthwhile to conclusively test the robustness of our findings.

## 5. Conclusions

Results from motor-cognitive testing do not correlate substantially with traditional assessments of cognitive and motor function. Integrative measurement tools may hence supplement the diagnostic spectrum used by health professionals and conditioning coaches in game sports. Yet, prior to their implementation, it should be tested if the test results can predict performance and injury risk.

## Figures and Tables

**Table 1:** Items of the motor-cognitive testing battery.

Test Item	Set-Up	Task	Outcome
Reaction UE	Stance in front of table with one sensor. Palm of dominant hand besides sensor.	Deactivate light with DH when flashing (20 lights).	Mean RT [s]
Reaction LE	One sensor on ground in front of standing participant.	Deactivate light with DF when flashing (20 lights).	Mean RT [s]
Choice-Reaction UE	Stance in front of table with 8 sensors arranged semicircular and equidistantly.	Deactivate randomly flashing lights with both hands (45 s).	Mean RT [s]
Choice-Reaction LE	8 sensors arranged semicircular and equidistantly in front of standing participant.	Deactivate randomly flashing with both feet (45 s).	Mean RT [s]
Stop-signal UE	Stance in front of table with one sensor. Side of dominant hand positioned next to sensor.	Deactivate light with DH when flashing dark blue (go, *n* = 50 lights), but not when light blue (no-go, *n* = 10 lights).	Mean RT [s], Errors [n]
Stop-signal LE	One sensor positioned on ground lateral to dominant leg of standing participant.	Deactivate light with DF when flashing dark blue (go, *n* = 42 lights), but not when light blue (no-go, *n* = 8 lights).	Mean RT [s], Errors [n]
Memory Span	Sensors (3 to 8, starting with 3, from there increasing by one) flexibly arranged on table.	Arrange sensors in order of flashing. Two trials per light count. Test ends if both are failed for the first time.	Time needed [s], sum score (pts.)
Reactive Agility A	8 sensors attached to the top of 8 maze-like arranged cones.	Out of two flashing lights, deactivate partials (outer ring only), ignore completes (center and ring). 24 light pairs.	Time needed [s]
Reactive Agility B	8 sensors attached to the top of 8 maze-like arranged cones.	Alternatingly deactivate completely and partially lighting sensors (two lighting at a time as in part A). 24 light pairs.	Time needed [s]
Run-Decide	Three sensors attached to wall at head level (two lateral, one central). Participant at 7.5 m distance from wall.	Run towards wall (4 m/s). At 3.5 m distance, cut to deactivate lateral sensors (first green light, than red light). If central sensor lights, reverse order of deactivation (10 runs, 4 with center light).	Time needed to deactivate first lateral sensor [s], errors [n]

For more details and illustration of the MC tests, please see Wilke et al. [11] UE = upper extremity, LW = lower extremity, DH = dominant hand, DF = dominant foot, s = seconds, pts. = points.

**Table 2:** Motor-cognitive testing items and assumed correlations with general cognitive and motor outcomes.

Test Item	Motor Predictors	Cognitive Predictors
Reaction UE	Sprint time	Stroop word/colour
Reaction LE	Sprint time, Dynamic balance	Stroop word/colour
Choice-Reaction UE	Sprint time	Stroop word/colour, TMT A/B
Choice-Reaction LE	Sprint time, Dynamic balance	Stroop word/colour, TMT A/B
Stop-signal UE	Sprint time	Stroop word/colour/colour-word
Stop-signal LE	Sprint time, Dynamic balance	Stroop word/colour/colour-word
Memory Span	Sprint time (time needed)	DS, TMT B, Stroop colour (time and errors)
Reactive Agility A	Sprint time, CMJ, Dynamic balance	DS, TMT A/B, Stroop word/colour/colour-word
Reactive Agility B	Sprint time, CMJ, Dynamic balance	DS, TMT A/B, Stroop word/colour/colour-word
Run-Decide	Sprint time, CMJ, Dynamic balance	DS, TMT A/B, Stroop word/colour/colour-word

UE = upper extremity, LW = lower extremity, CMJ = counter-movement jump, TMT = Trail Making Test, DS = Digit Span Test.
